# A Qualitative Study on Coping Strategies of Chinese Women With Metastatic Breast Cancer Undergoing Chemotherapy

**DOI:** 10.3389/fpsyg.2022.841963

**Published:** 2022-03-11

**Authors:** Yi-Qiang Guo, Qing-Mei Ju, Miaoning You, Azlina Yusuf, Ying Wu, Lean Keng Soon

**Affiliations:** ^1^School of Nursing, Capital Medical University, Beijing, China; ^2^Emergency General Hospital, Beijing, China; ^3^Key Laboratory of Carcinogenesis and Translational Research (Ministry of Education/Beijing), Department of Breast Oncology, Peking University Cancer Hospital & Institute, Beijing, China; ^4^School of Health Sciences, Universiti Sains Malaysia, Kelantan, Malaysia

**Keywords:** metastatic breast cancer women, chemotherapy, coping strategies, qualitative, future perspectives

## Abstract

**Objectives:**

Women who underwent chemotherapy (CT) for metastatic breast cancer (MBC) used both adaptive and maladaptive coping strategies but had low implementation levels. The present study explores the qualitative experience of coping strategies for women with MBC undergoing CT in Beijing.

**Methods:**

A hermeneutic phenomenological approach was employed on twenty Chinese MBC women undergoing CT. These interviews were transcribed verbatim, coded using thematic analysis, and analyzed using NVivo 11.

**Results:**

Three themes are highlighted: Maintaining hope; Spiritual growth, and Self-perceived support resources.

**Conclusion:**

The present study results have led to a greater understanding of the tremendous impact of CT on MBC women’s lives. This research provides insight into the scope of maintaining hope. Spiritual growth and self-perceived support resources were crucial factors to coping strategies among MBC women to improve their quality of life.

**Clinical Relevance:**

By attaining the adaptive coping strategies and further understanding about Chinese MBC women, health care professionals are encouraged to appraise MBC women’s specific problems and adopt effective interventions to improve MBC women’s psychosocial wellbeing.

## Introduction

Global breast cancer (BC) is the most common cancer among females (Sung et al., 2021). It accounts for about 30% of female cancers and has a mortality-to-incidence ratio of 15%, as the first oncological cause of death in women (Siegel et al., 2020). Moreover, BC is the most prevalent malignant tumor in Chinese women, there were 416,371 new cases of breast cancer among new-onset cancers, which accounted for 19.9% (World Health Organization, 2020), with rising occurrence rates (Cao et al., 2021). Furthermore, more than 30% of BC survivors diagnosed with early-stage cancer will eventually develop metastatic illness (Lu et al., 2009; Redig and McAllister, 2013). Although MBC women’s median survival time is 3 years, the range of overall survival time is more comprehensive because MBC survivors carry the illness for an extended period (Cardoso et al., 2020). Hence, the population of MBC survivors, who would undergo a long anti-cancer journey, is uprising.

Due to the complexity of advanced breast cancer, it can be a single site, a single nidus, multiple sites, or multiple niduses. Therefore, there are many treatment methods, including systemic treatment such as chemotherapy, endocrine therapy, targeted therapy, and immunotherapy, as well as topical therapy such as radiotherapy, surgery, radiofrequency ablation, and interventional treatment. These treatments can benefit patients and effectively prolong the survival time of patients. But currently, chemotherapy (CT) is still the most commonly used (Breast Disease Research Center of China Medical Women’s Association, 2020).

MBC patients suffer negative physical, psychological, and social impacts of cancer treatment on top of the shock, fear, anxiety, and uncontrollable feelings of uncertainty and mortality evoked when they receive the MBC diagnosis, and psychological distress was high among MBC women (Mishra and Saranath, 2019; Cardoso et al., 2020; Gradishar et al., 2020). Previous studies have identified various coping strategies used among MBC women to confront their life-threatening illness and lengthy and strenuous treatment, including decision-making. However, emotional support was the most frequently coping style utilized by newly diagnosed incurable cancer patients (Nipp et al., 2016; Calderon et al., 2021). And, denial coping is common when people are diagnosed with a terminal illness; it can be either protective or harmful in managing the health problem. Utilizing denial coping as the first step in dealing with life-threatening illness allows individuals to adjust to the situation. However, denial coping can become a problem when it persists and prevents individuals from adapting to the truth and receiving treatment (Pishori, 2013). Trevino et al. (2012) pointed out that accepting the fact and seeking support are the most frequent coping methods young MBC women adopt. Problem-based coping, emotion-based coping, and maladjustment were too general to describe MBC patients’ coping styles (Guo et al., 2017). Ahmadi (2015) revealed that cancer patients in Sweden preferred to use meaning-making coping methods, especially nature and a sense of self-control, to adjust themselves to the disease.

Most of MBC women actively engage with this condition and its therapy; nonetheless, they occasionally need to drift apart from this illness. Therefore, their coping style changes depending on the time or environment, and effective coping strategies for MBC women are complicated. Therefore, the research objective of the current study was to explore the qualitative experience of coping strategies of MBC women who were receiving CT in Beijing. The knowledge from this study can be used when informing MBC patients of possible coping strategies.

## Materials and Methods

### Design

The present study was performed using the hermeneutic phenomenological approach. Phenomenology is a term in qualitative research that refers to an interest in understanding phenomena from the actors’ viewpoints and depicting the world as it is experienced by the informants, based on the idea that reality is what people perceive (Kvale and Brinkmann, 2009). The selected phenomenon in this study was the lived experience of MBC women undergoing CT. Phenomenology is an appropriate method for accessing the world of these MBC women and understanding their true experiences and living with MBC. The hermeneutic approach is founded on the premise that all human consciousness is interpretative. As a result, it is possible to examine the participants’ experiences in the context of a psychosocial viewpoint on health and illness (Kvale and Brinkmann, 2009; Svenaeus, 2011). This strategy creates rigorous and consistent procedures of the ethical components of daily life that are difficult to obtain using traditional research methodologies. In the current study, we sought to explore the coping strategies for women with MBC undergoing CT. Such study places a heavy emphasis on reflective interpretation, as Heidegger demonstrated when he claimed that description and interpretation are inexorably intertwined (Edmonds and Kennedy, 2017). Comprehension develops during the study process, based on both the participant’s and the researcher’s prior understandings and the context (Gubrium, 2012).

### Participants

Participants were MBC patients from the Cancer Hospital in Beijing within two months in 2019. The participants were recruited through purposeful sampling. Inclusion criteria were: (i) diagnosed with MBC and undergoing chemotherapy and (ii) aged 18–70 years. Exclusion criteria were: (1) MBC women with mental health illness, (ii) severe comorbidity, (iii) Karnofsky’s Performance Status of ≤50, and (iv) declined participation. Sampling continued until data saturation (*n* = 20) was reached. Thus, the sample size was 5–25 in the acceptable phenomenology range.

### Ethical Considerations

The study was reviewed and approved by the Human Research Ethics Committee Beijing Cancer Hospital (Reference Number: 2018KT106) and conducted according to the Declaration of Helsinki and institutional guidelines. Participants were assigned pseudonyms during data reporting to protect subject anonymity and ensure confidentiality. Pseudonyms were used during data reporting to protect subject anonymity and confidentiality. For participants who agreed to participate, the study’s aim and objectives were explained thoroughly by the researchers. In addition, information sheets were provided to the participants for further clarification about the study. Participants were informed about their rights, and their agreement to participate in the study is considered written consent. The health status of participants was checked with Karnofsky’s Performance Status (KPS) given the anticipation of worse conditions during the interview. The KPS is an assessment tool for functional impairment and to assess the patient’s health status. Participants with a KPS of 50 or less or terminally ill were considered ineligible to be recruited to the study.

### Procedures

The first researcher, a nursing lecturer, developed the in-depth semi-structured interviews in English and Mandarin. The interviews were based on adaptive coping, a model that highlights the development of positive coping (Lazarus and Folkman, 1984; Folkman and Greer, 2000) and an extensive literature review to obtain the required data, including issues of concern raised by MBC patients. This model guided the study throughout, particularly the conceptualization of the interview guide and interpretation of data. The interviews consist of views on MBC and CT, experiences on MBC and CT, side-effects of CT, and coping with MBC and CT. The researchers (two nursing and midwifery lecturers, one nurse clinician, and two breast consultants’ experts) reviewed and double-checked the initial draft of the interview subject guide. The goal was to ensure that the interviews reflected the local context. The modified interview topic guide was pretested by conducting pilot interviews with six MBC patients in Mandarin to ensure it was understandable and useful in retrieving the required information. The interviews were done individually and face to face. After feedback from 6 MBC participants and experts who piloted the interview guide, the refined open-ended questions were used with prompts. For example, “What is your understanding of coping strategies when faced with disease spread? Can you tell me more about that?” to facilitate participants’ disclosure of coping strategies.

The researchers observed several procedures to ensure research rigor. First, the purposive sample technique was adopted in this study to select participants, the MBC patients undergoing CT. Thus, enabled the researchers to describe the phenomenon under study in all its nuances. Second, to ensure data trustworthiness, the researchers observed the following key components: credibility, transferability, dependability, and confirmability. Before study commencement, the first researcher addressed credibility by approaching participants who met the inclusion criteria. The purpose was to establish a good rapport with participants and explain the importance of the study. Third, the researchers called and arranged interviews with participants at times suitable for them. Face-to-face, semi-structured interviews were scheduled and conducted in Mandarin by the first researcher at the convenience of the participants. The interviews were conducted in a guest room with a quiet and private atmosphere while participants waited for therapy treatment. All participants were given enough time to share their feelings and experiences during data collection fully. Each interview was digitally recorded, lasted for 45–60 min, and audio-recorded with participants authorization.

To achieve transferability, responses from participants were probed where necessary during the interview to get clarity of responses and allow participants to give detailed descriptions of their MBC experiences fully. Finally, the researchers are responsible for ensuring that the data collected were complete, accurate, and well interpreted by reinstating the statement given by participants to clear any doubt. According to Lincoln and Guba (1985), the researcher’s responsibility is to provide thick descriptions so that readers can evaluate the data’s application in various situations and determine whether or not a transfer is feasible. Researchers ensure the research process is logical, traceable, and documented to achieve dependability (Noble and Smith, 2015). Researchers ensured the study findings denoted what the participants narrated when interviewed through verbatim quotes to establish confirmability (Noble and Smith, 2015). The findings must reflect the participants’ voices and the conditions of the inquiry and not the researcher’s biases. Therefore, biases and subjectivities were avoided in the study. Thus, established an audit trail of all the processes and validity of the finding. Finally, the criteria of data saturation, rather than setting a fixed sample size, was followed. Hence, it enables the researchers to stop inclusions only when the investigation phenomenon has been fully explored. The researcher continued collecting data until she was confident that data saturation had been attained.

### Data Analysis

A thematic analysis (Boyatzis, 1998) was performed. The transcripts were transcribed verbatim into written Chinese and translated into English. Data analysis was undertaken concurrently with data collection. A thematic analysis was to tease out the transcripts, in which four researchers repeatedly read along with listening to the audio recordings. Further, discussions were made within the multidisciplinary research team. Thus, ensure the validity and inter-subjectivity of the analytic process. The research team discussed the broader initial codes to generate and name categories and condense subthemes and themes until they reached a consensus. Following this, the researcher and a bilingual expert in the research team carried out the English translation of participant quotations. Credibility was operationalized through member checking, which involves testing the results and interpretations with MBC participants (Lincoln and Guba, 1985). During the data analysis, special emphasis was paid to negative examples to incorporate the participants’ convergent and divergent voices. The researchers followed the unified criteria for reporting qualitative research checklist to guarantee that the study report satisfied high methodological standards. As a result, helps in verifying the study’s transferability and reliability (Lincoln and Guba, 1985). Reflexivity was maintained throughout the analysis process, returning to the data. Each researcher explained the personal categories for occasional cases where discrepancies or nuances existed. Following, all researchers returned to the original data to review the text and agree on the categories that represented the data. Furthermore, to verify the data were accurate, the interview transcripts were sent to participants until they expressed their agreement with the content.

## Results

Twenty MBC interviewees’ age ranged between 36 and 61 years, with an average of 46.75 ± 8.49. Seventeen were married, one was divorced, and two were widowed. The time between MBC diagnosis and participation in the study ranged from 0.4 months to 73.4 months, with a mean of 17.30 ± 18.43, while the median time was 9.75 months (Table 1).

**Table 1:** Socio-demographic characteristics of MBC women.

Name^*^	Age	Marital status	Education level	Metastatic site	Duration^a^
YY	58	Married	Junior college	Bone; Lung; Liver; Brain	73.4
WHF	44	Married	Junior college	Bone; Liver	23.7
JAT	51	Married	Secondary school	Peritoneum	13.2
PCY	37	Married	University	Bone; Liver	0.4
WLX	37	Married	University	Lung; Liver	5.7
TMX	53	Widowed	Secondary school	Liver	36.5
LYL	38	Married	Secondary school	Lung	8.2
WGX	57	Married	High school	Skin	3.4
BWL	44	Married	High school	Bone; Liver	4.1
LYF	46	Married	High school	Liver	35.4
TJZ	56	Married	High school	Bone; Liver	29.4
ZQY	44	Married	University	Liver	28.4
LH	36	Married	University	Bone; Lung	11.3
XLH	55	Divorced	Secondary school	Lung; Liver	39.3
LL	46	Married	Secondary school	Lung	1.3
LDH	61	Widowed	Secondary school	Bone	7.0
WWD	38	Married	University	Bone; Liver	11.6
DLF	38	Married	Secondary school	Bone; Liver	3.4
WQF	57	Married	High school	Liver	8.0
LJH	39	Married	Junior college	Lung	2.5

a *Months from diagnosis to interview date.*

* *All the names are pseudonyms.*

Emerging from this study were three main themes and nine subthemes described using excerpts from the MBC participants’ perspectives. The three major themes were: “Maintaining hope,” “Spiritual growth,” and “Self-perceived support resources.” Table 2 illustrates the emergent themes and subthemes.

**Table 2:** Synthesized themes and subthemes of Chinese MBC women’s coping strategies.

Themes	Subthemes
Maintaining hope	Internalized acceptance Intentional self-distraction from negative mood Expectations Set goals
Spiritual growth	Positive thinking Cherishing the current moment Seeking spiritual resources
Self-perceived support resources	Connectedness with intimate individuals Reliable health professionals

## Theme 1: Maintaining Hope

Inevitably, incurable MBC is associated with notions of death, no future, and hopelessness. Therefore, maintaining hope for MBC women’s survival is paramount. Maintain hope; internalized acceptance is the significant prerequisite. Setting appropriate goals and generating expectations were components that contributed to maintaining hope. The subthemes emanating from this theme are explained as follows.

When receiving the MBC diagnosis, MBC women’s mind-attack feeling came out. It was tough to accept the horrific news. As one woman narrated, “I did not want to know the illness-related information at early diagnosis phase.” (WWD).

### Internalized Acceptance

Almost all participants found it hard to accept the reality (of having MBC) in the short term. However, their comments indicate that internalized acceptance helped MBC women move on and cope with their situation. One participant who has been diagnosed with MBC for two months and undergoing CT narrated that:

*“I hope to have a happy, peaceful and well-functioning life and no discomfort. When I was diagnosed with cancer spread, my life was like a race against time. The doctor told me I had another 6 months to live. I have to fight hard. Half a year is my target survival time. I will try to achieve a longer time—possibly 1 year or 1.5 years. But if God wants to take me, I have no choice but to accept fate.” (LYF).*

### Intentional Self-Distraction From Negative Mood

After accepting the reality of MBC, participants explained that they were prone to negative emotions as a result of debilitating, strenuous treatment. They turned to self-distraction as a way of overcoming this. Some participants pointed out that they kept “busy” and used this self-distraction to reduce “dwelling” on and or thinking about the disease, a strategy that increased their coping ability. The following quotes illustrate this:

*“I keep myself busy like go to the stock market, watch TV, prepare meals for my daughter and grandson. Sometimes I sing and do not have time to think about trivial matters.” (TMX).*

*“I attend to group chorus and sing together with people in the park. What I can say is that it makes me feel great and happy.” (XLH).*

Among these MBC participants, many different sources of hope have been identified that look into the broad outlooks on life. Most looked forward to new, advanced medical treatments for their disease. Two participants reported:

*“I surf the internet every day for news that new drugs can cure cancer. I would tell myself to be tough to wait for the next year, and then I will be cured.” (XLH).*

*“I still conceive hope, even though I know the average survival time is around 2 years for a patient with my condition. From my husband’s life attitude, he always looks at the bright side. I should actively cope with the treatment and balance my emotion. Miracle exists, and this miracle represents hope.” (WLX).*

### Expectations

Despite most MBC women participants reporting acceptance of their reality, they expressed their expectations and wishes for the remainder of their lives and their deaths. For example, one of the participants narrated:

*“I think this disease makes me appreciate how to value life. I often ponder on life and death. Life gets harder in the last stage. I do not want to suffer too much pain or discomfort. Sometimes I think of my disease and wish for painless death [die peacefully] to end this torture.” (WLX).*

### Set Goals

As the illumination of the MBC women, they benefit from fostering expectations and setting short-term and achievable goals. Many of the participants expressed how they used goal setting to maintain hope. Most of them redirected their minds from their illness through in-depth thinking and finding achievable goals. For example:

*“Life has to move on. Despite the agony I felt, I have to be active and energetic to prepare food for my family. I will not let the disease beat me. Otherwise, my family members would lose confidence.” (LDH).*

## Theme 2: Spiritual Growth

Participants in this study reported spirituality as essential to their wellbeing. Spirituality included the purpose and value of their life in crisis and was associated with coping and treatment. They emphasized the importance of spirituality in helping them live with the disease and its spread during their painful cancer journey. It is summed up in one participant’s thoughts:

*“Diagnosing with cancer spread changes my way of looking. In this disease crisis, I had to adjust and strengthen my spiritual belief and health.”*

This theme was classified into three subthemes: positive thinking, cherishing the current moment, and seeking spiritual resources.

### Positive Thinking

Positive thinking played a pivotal role for MBC women. They stated that it helped reduce the stress, harm, and fear associated with the experience of cancer. One narrated that:

*“Although cancer spread to my liver, I had to cheer up and to cope. Having positive thinking was important. When I feel uncomfortable in my liver, the thought of the tumour shrink can make me in a better mood.” (WQF).*

Instead of sadness, some participants expressed that they used the “Q” spirit. In Chinese, the “Q” spirit or “Q” mentality means to enjoy what one has rather than focusing on what one will lose. They described the psychological victory as an illness to wellness coping strategy. For example:

*God creates everybody equally. I used the “Q” spirit to cope, and I feel better.” Compared to the superstar (Chinese singer Yao BeiNa) diagnosed with breast cancer, she is still single while I have a happy family with my lovely daughter. (LH).*

### Cherishing the Current Moment

Living in every moment reflects the participants’ view of MBC as causing them to live with finite time after the inherent deep thinking that they have an incurable illness. Therefore, they cherished every present moment and spent the valuable time they had left with no regrets. The following statements reflect two participants’ views:

*“Nowadays, I take health regimen such as exercise and eat healthy food every day. I do good to others and enjoy every beautiful moment.” (WWD).*

*“I do not want to think about the future in my heart. In fact, I dare not think about it. I continue to cherish my day as a routine.” (LJH).*

### Seeking Spiritual Resources

Spirituality arises for MBC women when facing a life crisis. For these women, spirituality is a dimension through which they can fight the sense of fear throughout their disease, as illustrated in the following participants’ comments:

*“I have this disease and need to live my life. I relate to God through my prayers and blessing, which makes me happy.” (ZQY).*

*“Death to me does not matter. My concern is my husband. If I die, life will get tough for him. He is old and alone. When this day comes, who will take care of him? Who will care about his activity of daily living and needs? What I wish now is pray for blessing and spend valuable time with my family, particularly with my husband before God take me, which make me feel peaceful and secure.” (TJZ).*

*“My most crucial change after diagnosis was faith in Buddhism, which spiritually grants inner peace to me and level down my negative mood. It sort of leads me to a focused and meaningful life while guiding me to look at the actual worldly dimension. For example, I have a sleep disorder, and reading mantras benefits me and sleep well. Meditation also helps relieve stress and anxiety, and I find it helps a lot.” (WLX).*

## Theme 3: Self-Perceived Support Resources

Self-perceived emotional support from family, especially spouses, and health professionals was essential in their ability to attain the confidence to pursue an anti-cancer trajectory. These effects were categorized into two subthemes: connected with intimate individuals and reliable health professionals.

### Connectedness With Intimate Individuals

Many MBC women appreciated their family members’ efforts in connectedness and providing them with emotional support. The following quote represents one MBC participant’s views:

*“I deeply realized this cancer-fighting process cannot [happen] without support from family, especially from my husband. It’s a long-term fighting process, test not only me but also test my whole family.” (LH).*

### Rely on Health Professionals

Participants reported that doctors and nurses were influential individuals who played vital roles in their illness trajectory and treatment; they saw them as instrumental in helping them to maintain their health. They treated the doctors as life-saving “straw.” The health professionals’ (especially physicians’) consolidative words and behavior helped calm their fears and give them the “heart” to undergo treatment and the whole journey, as expressed by one participant:

*“Doctors and nurses are the people who help me to cope with the disease. In my view, I rely on this group of health care staff.” (WQF).*

## Discussion

The results highlight that the MBC Chinese women participating in this research considered accepting their situation as an essential coping strategy that played a prerequisite role in the cancer-fighting process. After the acceptance, they can move forward to the future. Based on this inner acceptance, the women expressed their involvement in treatment decision-making and planning for expenditure. Accepting death as one component of the whole life journey instead of perceiving it as the opposite side of life was believed to help them cope with an incurable illness. In addition, they felt that keeping themselves busy could divert their attention from distress. This acceptance appears to arise from their attainment of a peaceful mind, proactive approaches, and hope for the future; once achieved, acceptance enabled the women to enter a place of self-transcendence above their approaching death. Consistent with a previous study undertaken by Secinti et al. (2019), the acceptance of life-threatening disease appears to be an important target in interventions to reduce general and cancer-specific distress and coping in cancer patients. People typically find optimism when they realize that they have cancer. Accepting is supported by Nipp et al. (2017) research, who found that accepting the reality of the situation, something that many of our participants emphasized.

People living with cancer require hope to fight the disease. Hope is crucial at every stage of human life’s growth and development. The MBC women acknowledged that the promise of living and returning to a normal life had fueled their will to heal and combat the disease efficiently. Researchers described that acceptance assisted participants to adopt coping mechanisms that were helpful to encouraging hope (Chinh et al., 2020). Maintaining hope can be a valuable asset in coping with an incurable illness. In this study, the Chinese MBC women suffered disease progression more than twice. Their narrative highlights the expectation of being cured, even though they knew the incurable reality and had accepted it. Still, they held onto hope. Our findings are congruent with Ginter (2020), where cancer patients with disease spread continued to hope for a cure, seen as a transition through different points along the illness trajectory and symptom control. However, Corner (2022) emphasize that people with MBC have a short amount of time left and assume it is an immediate death sentence, which can no longer be treated. Therefore, concurs with our study that offering unrealistic hope or being complicit in patients’ unrealistic expectations is not warranted. Influenced by China’s mainstream social and cultural values, most people do not talk about death. People have never thought about or integrated the relationship between life and death before. Hence, these feelings would stay for a long time in the stage of hoping for a cure. Future studies should facilitate patients reconstructing the style of hope and offer one another expectation.

The expectation of being cured was one way the MBC women in this study maintained hope. Meanwhile, they demonstrated a range of strategies to cope with the reality of inevitable death, such as refocusing their goals, keeping themselves busy, and immersing themselves in everyday activities. Participants highlighted how self-distraction as a coping strategy was helpful when they felt down or depressed. Optimizing their coping strategy, broadening achievable life goals, enhancing the will to achieve goals, and facilitating the plan-making process were essential factors in helping them maintain hope. The MBC women expressed that setting and achieving goals resulted in increased resilience. Our results concur with recent literature that has found a clear link between dealing with engendering hope (Broadhurst and Harrington, 2016). Therefore, encouraging MBC women to identify and attain goals to maintain hope may help them optimize coping during the illness trajectory and treatment. We found that the majority of the MBC women participants reported using adaptive coping strategies, including maintaining hope, positive thinking, spiritual growth, strengthening family ties and love and self-perceived support resources. These are thought to impact both qualities of life and survival undergoing CT. However, the current study found that some MBC women participants used maladaptive coping techniques, including self-distraction rather than adaptive coping mechanisms. The above finding is congruent with those of Dev et al. (2021), who reported that patients with chronic diseases (such as cancer) displayed positive and problem-focused coping styles less than other coping styles. Participants who may be more likely to use maladaptive coping strategies may benefit from further psychological support. Our findings highlight that MBC women using maladaptive coping strategies felt helpful when down or depressed. Therefore, maladaptive coping strategy self-distraction can have a significant impact to encourage MBC women to combat psychological distress. The findings of the current study have practical implications for health care management that maladaptive coping can have a role in decreasing depression.

One of the coping strategies that MBC women use to curtail their stress is positive thinking concurs with the results of a study that evaluated the coping strategies BC women used to deal with cancer-related psychological problems (Youll and Meekosha, 2011; Yusuf et al., 2013). Problem-focused coping strategies, such as cognitive acceptance and positive thinking, were mostly considered beneficial (Anusasananun et al., 2013). This study indicated that trying to be hopeful and have a positive perspective have very important roles among MBC women. Similarly, in the study reported in this paper, positive thinking encouraged MBC women to optimize their perceptions of how they feel about life and medical treatment and guide them toward setting and achieving goals (Trompetter et al., 2022). Our results also concur with Ahmadi et al.’s (2016) study. Thus, participants fighting cancer will use positive-life-perspective coping strategies by interpreting the situation *via* an optimistic pathway oy strengthening their feelings of self-responsibility. Therefore, reframing positive thinking as an efficient coping strategy should be promoted among MBC women to inspire motivation to participate in life and daily activities.

As this study’s participants did, living in the present, which means cherishing current life and happiness, is a strategy confirmed by Lam et al. (2016). In Lam et al.’s study, patients with low stable or transient distress were prone to cherish the present moment as one pathway to coping adaptively compared to patients suffering from long-term psychological distress. Relishing the moment instead of dwelling on the past or looking to the future is a common theme among the current study, Lam et al. (2016), and Li et al. (2015), who found that each day lived represented an extra day the cancer patient and their family had earned.

Participants in this study chose to reduce negative thoughts or impacts from the illness to get the most out of every moment. This finding concurs with other research, which has found that living in the present moment enables cancer patients to maintain a positive perspective (Pacsi, 2015). In addition, developing positive thoughts can decrease anxiety and depression. Our findings also concur with other literature about coping with incurable or chronic conditions (Lewis et al., 2016).

There is an assumption that believing in “spiritual life” can reduce the fear of facing death (Bower et al., 2015; Puchalski et al., 2018; Garduño-Ortega et al., 2021). While China is not a religious country, many Chinese people are spiritual and incorporate Confucianism, Taoism, and Buddhism into their daily lives (Huang and You, 2015; Pahlevan Sharif et al., 2021). The study illustrated by the MBC women participants’ deep reflection on the value of life arose from their experience of the misery inflicted by their cancer journey, a source of inner strength and peacefulness. In addition, Connectedness to intimate others (usually their spouse, children, or other family members) played a pivotal role in satisfying their spirituality. MBC participants’ revelation that the need to enjoy daily life with their intimate spouse or family members, constant thinking of the impact of disease spread pending death, echoes the importance of cherishing time spent with close family members, goodness and love concur with previous studies (Young et al., 2015; Mishra and Saranath, 2019). The current study’s findings also correlate with Broadhurst and Harrington’s (2016) study, which found that strengthening family ties and love is associated with engendering hope. In addition, Corn et al. (2020) found that hope has a solid connection to spiritual wellbeing and spiritual needs and health-promoting behavior. Therefore, it is pertinent for nurses and other healthcare professionals involved in cancer care and treatment to meet the spiritual needs of MBC patients.

In this study, the MBC women identified self-perceived support resources, such as support from family members, friends, and reliable health professionals, as essential for giving them the confidence to cope with their dire situation. These findings back up Kugbey et al.’s (2020) research on women living with BC in Ghana, which found that social support is one of the most important coping strategies for patients dealing with emotional and psychological issues. In addition, concerning health professionals, MBC women in the current study perceived them as playing a positive role in nurturing and maintaining hope. The MBC women appraised self-perceived support resources as a coping strategy to balance the stresses stemming from their illness/treatment advent. Accordingly, people with breast cancer who have greater social support cope better with their illness (Yeung and Lu, 2018). Therefore, helping MBC women cope with the disease and treatment effectively needs health professionals to use empathy, observe and understand the MBC women timely, endeavor to balance their minds, and perceive the support resources. The present findings highlight the importance of improved social networks and support systems for women living with MBC to protect them from the myriad problems that come with living with MBC.

### Limitations and Future Research

This research has been conducted on Chinese MBC women and in one cancer hospital in China and cannot be generalized for the MBC women of other countries. Nevertheless, it is worth noting that the experiences of Chinese MBC women have been generally explored. However, this study did not explore MBC women’s perceptions of the treatment decision-making processes. Therefore, it is recommended to perform similar studies on the MBC women from other religions and countries; and cancer treatment decision-making processes.

## Conclusion

The insight this study provides into the culture-based predisposition of Chinese women to adopt specific coping strategies in the face of MBC adds a valuable resource to an area that has not been adequately studied. This understanding of what coping strategies are effective for Chinese MBC women can help health care teams develop culturally tailored interventions for such women and other MBC women, taking cultural differences into account. The researchers contend that the new knowledge provided here will contribute to less negative impacts of MBC and its treatment and a higher QoL for MBC women in China and elsewhere.

### Implications for Practice

Knowing that maintaining hope and developing spiritual growth can improve MBC women’s QoL by increasing their inner peace can help MBC patients to adopt coping strategies during their illness. It is essential to enhance MBC women’s spiritual growth by recognizing the meaning of intimate relationships and self-value. Timing for interventions to increase their spirituality must be evaluated individually, at a time when each woman is eager to talk. Given this study’s findings and findings from the literature, it is suggested that all health professionals, but especially nurses, provide the hope, social support, and spiritual help required by MBC women. Nurses are indicated as the most likely health professionals to undertake this.

## Data Availability Statement

The original contributions presented in the study are included in the article/Supplementary Material, further inquiries can be directed to the corresponding author.

## Ethics Statement

The studies involving human participants were reviewed and approved by Medical Ethics Committee of Beijing Cancer Hospital. The patients/participants provided their written informed consent to participate in this study. Written informed consent was obtained from the individual(s) for the publication of any potentially identifiable images or data included in this article.

## Author Contributions

YQG, SLK and YW: conceptualization of the study. YQG, AY, QMJ and MY: methodology. YQG, QMJ and MY: investigation. YQG, QMJ, YW, MY and SLK: formal analysis. YQG: drafting the work. SLK, AY and YW: revised it critically for important intellectual content. YW, SLK and AY: supervision. All authors contributed to the article and approved the submitted version.

## Funding

This study is supported by a project granted by the Beijing Postdoctoral Research Foundation (Grant Number 2020-ZZ-013).

## Conflict of Interest

The authors declare that the research was conducted in the absence of any commercial or financial relationships that could be construed as a potential conflict of interest.

## Publisher’s Note

All claims expressed in this article are solely those of the authors and do not necessarily represent those of their affiliated organizations, or those of the publisher, the editors and the reviewers. Any product that may be evaluated in this article, or claim that may be made by its manufacturer, is not guaranteed or endorsed by the publisher.
